# *catena*-Poly[[diaqua­[(4-tolyl­sulfan­yl)acetato-κ*O*]cadmium(II)]-μ-4,4′-bipyridine-κ^2^ *N*:*N*′]

**DOI:** 10.1107/S1600536808024720

**Published:** 2008-08-06

**Authors:** Wen-Xuan Cai, Xiao-Yong Zheng, Xiao-Hong Geng, Yun-Long Feng

**Affiliations:** a Zhejiang Key Laboratory for Reactive Chemistry on Solid Surfaces, Institute of Physical Chemistry, Zhejiang Normal University, Jinhua, Zhejiang 321004, People’s Republic of China

## Abstract

The title complex, [Cd(C_9_H_9_O_2_S)_2_(C_10_H_8_N_2_)(H_2_O)_2_]_*n*_, has a linear chain structure. The central Cd^II^ ion is in a slightly disorted octa­hedral environment, coordinated by two aqua ligands, two (4-tolyl­sulfan­yl)acetate ligands and two bridging 4,4′-bipyridine ligands. The Cd^II^ ion lies on a twofold rotation axis. Inter­molecular O—H⋯O hydrogen bonds connect adjacent chains, forming a layer structure. An intramolecular O—H⋯O hydrogen bond is also present.

## Related literature

For related literature, see: Lin *et al.* (2006 ▶); Zheng *et al.* (2006 ▶). 
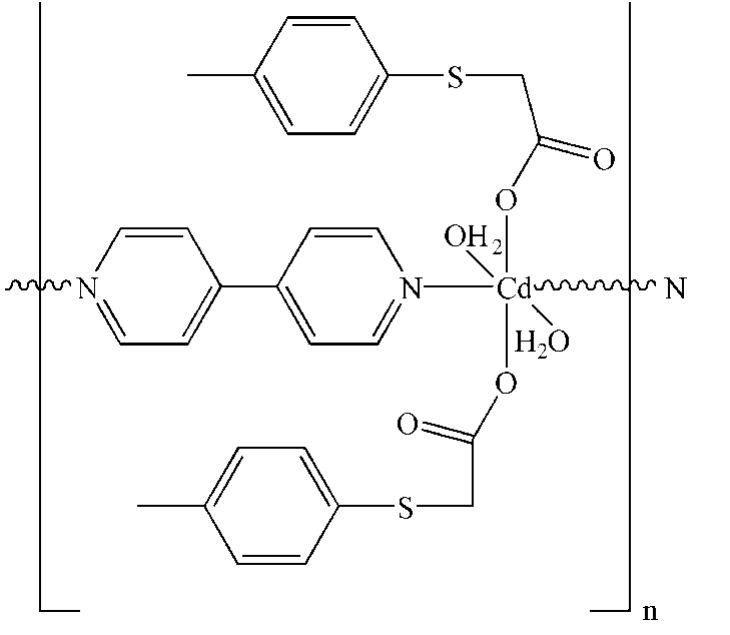


## Experimental

### 

#### Crystal data

[Cd(C_9_H_9_O_2_S)_2_(C_10_H_8_N_2_)(H_2_O)_2_]
*M* *_r_* = 667.09
Monoclinic, 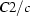

*a* = 21.659 (4) Å
*b* = 11.590 (2) Å
*c* = 11.137 (2) Å
β = 93.88 (3)°
*V* = 2789.3 (9) Å^3^
*Z* = 4
Mo *K*α radiation
μ = 0.98 mm^−1^
*T* = 296 (2) K
0.40 × 0.35 × 0.17 mm

#### Data collection

Bruker APEXII area-detector diffractometer
Absorption correction: multi-scan (*SADABS*; Sheldrick, 1996 ▶) *T*_min_ = 0.68, *T*_max_ = 0.85
12185 measured reflections
3154 independent reflections
2937 reflections with *I* > 2σ(*I*)
*R*_int_ = 0.017

#### Refinement

*R*[*F* ^2^ > 2σ(*F* ^2^)] = 0.032
*wR*(*F* ^2^) = 0.112
*S* = 1.12
3154 reflections
184 parameters
3 restraints
H atoms treated by a mixture of independent and constrained refinement
Δρ_max_ = 0.60 e Å^−3^
Δρ_min_ = −0.37 e Å^−3^

### 

Data collection: *APEX2* (Bruker, 2006 ▶); cell refinement: *SAINT* (Bruker, 2006 ▶); data reduction: *SAINT*; program(s) used to solve structure: *SHELXS97* (Sheldrick, 2008 ▶); program(s) used to refine structure: *SHELXL97* (Sheldrick, 2008 ▶); molecular graphics: *SHELXTL* (Sheldrick, 2008 ▶); software used to prepare material for publication: *SHELXTL*.

## Supplementary Material

Crystal structure: contains datablocks I, global. DOI: 10.1107/S1600536808024720/at2595sup1.cif

Structure factors: contains datablocks I. DOI: 10.1107/S1600536808024720/at2595Isup2.hkl

Additional supplementary materials:  crystallographic information; 3D view; checkCIF report

## Figures and Tables

**Table 1:** Hydrogen-bond geometry (Å, °)

*D*—H⋯*A*	*D*—H	H⋯*A*	*D*⋯*A*	*D*—H⋯*A*
O1*W*—H1*WA*⋯O2	0.82	1.94	2.667 (4)	148
O1*W*—H1*WB*⋯O1^i^	0.805 (17)	2.04 (2)	2.782 (3)	154 (4)

Symmetry code: (i) 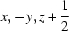.

**Fig. 1.**
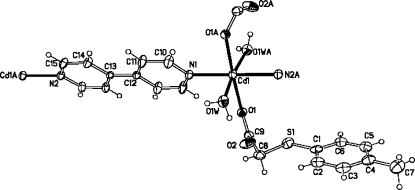
A view of part of the title structure, showing 30% probability displacement ellipsoids. [Symmetry codes: (a) -x, y, -z+1/2; (b) x, y+1, z; (c) x, y-1, z.]

**Fig. 2.**
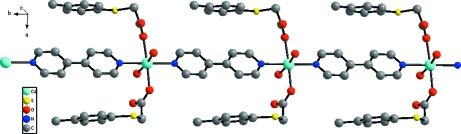
The chain structure of the title compound. All H atoms have been omitted for clarity.

## supplementary crystallographic information

### Comment

The title compound, (I), is isostructural with the Ni^II^ (Lin *et al.*, 2006) and Co^II^ analogues (Zheng *et al.*, 2006). The structure of (I) (Fig.1) consists of linear chains formed through 4,4'-bipy ligands linking six-coordinated Cd^II^ ions which lie on twofold rotation axes. Intermolecular O—H···O hydrogen bonds link neighboring chains to form a two-dimensional network. It is notable that these chains are arranged alternately and the 4-tolysulfanyl groups are almost coplanar. There is no signifcant π-π interactions between the planes of adjacent chains with centroid-centroid distance of 6.19 (1)Å and plane-to-plane distance of 3.64 (1) Å.

### Experimental

CdSO_4_.8/3H_2_O (0.128 g, 0.5 mmol), (4-tolylsulfanyl)acetic acid (0.091 g, 0.5 mmol), 4,4'-bipy (0.039 g, 0.25 mmol) and H_2_O (18 ml) were sealed in a 25 ml stainless-steel reactor with a Teflon-lined stainless steel reactor and the solution was heated at 433 K for 72 h and then cooled to room temperature over a period of 72 h. Colourless crystals suitable for X-ray analysis were obtained.

### Refinement

The methyl groups were allowed to rotate to fit the electron density [C—H = 0.96 Å and *U*_iso_(H) = 1.5*U*_eq_(C)]; the other H atoms were positioned geometrically [aromatic C—H = 0.93 Å and aliphatic C—H = 0.97 Å, *U*_iso_(H) = 1.2*U*_eq_(C)]. Water H atom H1WA was positioned geometrically, with O—H = 0.82 Å, and the other water H atoms H1WB was located from a difference Fourier map, and they were refined with distance restraints of O—H = 0.85 (2) Å and H···H = 1.30 (2) Å; their displacement parameters were set to 1.5*U*_eq_(O).

### Crystal data

**Table:**

[Cd(C_9_H_9_O_2_S)_2_(C_10_H_8_N_2_)(H_2_O)_2_]	*F*_000_ = 1360
*M**_r_* = 667.09	*D*_x_ = 1.589 Mg m^−^^3^
Monoclinic, *C*2/*c*	Mo *K*α radiation λ = 0.71073 Å
Hall symbol: -C 2yc	Cell parameters from 7745 reflections
*a* = 21.659 (4) Å	θ = 2.7–27.5º
*b* = 11.590 (2) Å	µ = 0.98 mm^−^^1^
*c* = 11.137 (2) Å	*T* = 296 (2) K
β = 93.88 (3)º	Block, colourless
*V* = 2789.3 (9) Å^3^	0.40 × 0.35 × 0.17 mm
*Z* = 4	

### Data collection

**Table:**

Bruker APEXII area-detector diffractometer	3154 independent reflections
Radiation source: fine-focus sealed tube	2937 reflections with *I* > 2σ(*I*)
Monochromator: graphite	*R*_int_ = 0.017
*T* = 296(2) K	θ_max_ = 27.5º
ω scans	θ_min_ = 2.7º
Absorption correction: multi-scan(SADABS; Sheldrick, 1996)	*h* = −27→27
*T*_min_ = 0.68, *T*_max_ = 0.85	*k* = −14→14
12185 measured reflections	*l* = −14→14

### Refinement

**Table:**

Refinement on *F*^2^	Secondary atom site location: difference Fourier map
Least-squares matrix: full	Hydrogen site location: inferred from neighbouring sites
*R*[*F*^2^ > 2σ(*F*^2^)] = 0.032	H atoms treated by a mixture of independent and constrained refinement
*wR*(*F*^2^) = 0.112	*w* = 1/[σ^2^(*F*_o_^2^) + (0.0658*P*)^2^ + 5.8321*P*] where *P* = (*F*_o_^2^ + 2*F*_c_^2^)/3
*S* = 1.12	(Δ/σ)_max_ < 0.001
3154 reflections	Δρ_max_ = 0.60 e Å^−^^3^
184 parameters	Δρ_min_ = −0.37 e Å^−^^3^
3 restraints	Extinction correction: none
Primary atom site location: structure-invariant direct methods	

### Special details

**Table:**

Geometry. All e.s.d.'s (except the e.s.d. in the dihedral angle between two l.s. planes) are estimated using the full covariance matrix. The cell e.s.d.'s are taken into account individually in the estimation of e.s.d.'s in distances, angles and torsion angles; correlations between e.s.d.'s in cell parameters are only used when they are defined by crystal symmetry. An approximate (isotropic) treatment of cell e.s.d.'s is used for estimating e.s.d.'s involving l.s. planes.
Refinement. Refinement of *F*^2^ against ALL reflections. The weighted *R*-factor *wR* and goodness of fit *S* are based on *F*^2^, conventional *R*-factors *R* are based on *F*, with *F* set to zero for negative *F*^2^. The threshold expression of *F*^2^ > σ(*F*^2^) is used only for calculating *R*-factors(gt) *etc*. and is not relevant to the choice of reflections for refinement. *R*-factors based on *F*^2^ are statistically about twice as large as those based on *F*, and *R*- factors based on ALL data will be even larger.

### Fractional atomic coordinates and isotropic or equivalent isotropic displacement parameters (Å^2^)

**Table:**

	*x*	*y*	*z*	*U*_iso_*/*U*_eq_	
Cd1	0.0000	0.05131 (2)	0.2500	0.03198 (12)	
S1	0.14880 (4)	0.21253 (9)	−0.05125 (7)	0.0514 (2)	
O1	0.08144 (10)	0.05635 (16)	0.1325 (2)	0.0353 (5)	
O1W	0.06445 (12)	0.0644 (2)	0.4169 (2)	0.0415 (5)	
H1WA	0.0990	0.0836	0.3982	0.062*	
H1WB	0.0700 (14)	0.012 (3)	0.464 (3)	0.047 (11)*	
O2	0.15198 (12)	0.1218 (3)	0.2705 (2)	0.0632 (8)	
N1	0.0000	−0.1422 (3)	0.2500	0.0290 (6)	
N2	0.0000	−0.7537 (3)	0.2500	0.0303 (7)	
C1	0.16190 (13)	0.3534 (3)	0.0059 (3)	0.0422 (7)	
C2	0.19048 (16)	0.3817 (4)	0.1177 (3)	0.0506 (9)	
H2A	0.2025	0.3234	0.1717	0.061*	
C3	0.20102 (17)	0.4945 (4)	0.1488 (3)	0.0544 (9)	
H3A	0.2210	0.5109	0.2234	0.065*	
C4	0.18302 (16)	0.5854 (4)	0.0730 (3)	0.0520 (8)	
C5	0.15375 (18)	0.5566 (3)	−0.0382 (3)	0.0508 (9)	
H5A	0.1407	0.6152	−0.0910	0.061*	
C6	0.14372 (16)	0.4440 (3)	−0.0715 (3)	0.0469 (9)	
H6A	0.1245	0.4275	−0.1468	0.056*	
C7	0.1952 (2)	0.7075 (5)	0.1109 (4)	0.0714 (12)	
H7A	0.2389	0.7186	0.1271	0.107*	
H7B	0.1742	0.7236	0.1822	0.107*	
H7C	0.1803	0.7586	0.0476	0.107*	
C8	0.17850 (15)	0.1165 (4)	0.0670 (3)	0.0495 (8)	
H8A	0.1879	0.0426	0.0316	0.059*	
H8B	0.2169	0.1481	0.1029	0.059*	
C9	0.13410 (13)	0.0967 (3)	0.1664 (3)	0.0385 (6)	
C10	−0.02550 (18)	−0.2021 (3)	0.3350 (3)	0.0470 (8)	
H10A	−0.0435	−0.1619	0.3959	0.056*	
C11	−0.02676 (18)	−0.3207 (3)	0.3379 (3)	0.0441 (8)	
H11A	−0.0457	−0.3584	0.3992	0.053*	
C12	0.0000	−0.3838 (3)	0.2500	0.0259 (7)	
C13	0.0000	−0.5111 (3)	0.2500	0.0250 (7)	
C14	−0.02533 (15)	−0.5744 (3)	0.3404 (3)	0.0357 (6)	
H14A	−0.0432	−0.5363	0.4029	0.043*	
C15	−0.02423 (15)	−0.6931 (3)	0.3382 (3)	0.0365 (6)	
H15A	−0.0410	−0.7331	0.4006	0.044*	

### Atomic displacement parameters (Å^2^)

**Table:**

	*U*^11^	*U*^22^	*U*^33^	*U*^12^	*U*^13^	*U*^23^
Cd1	0.03971 (19)	0.02551 (18)	0.03160 (18)	0.000	0.00893 (12)	0.000
S1	0.0526 (5)	0.0682 (6)	0.0343 (4)	−0.0107 (4)	0.0090 (3)	0.0052 (4)
O1	0.0353 (11)	0.0355 (11)	0.0362 (11)	−0.0019 (7)	0.0104 (9)	−0.0040 (8)
O1W	0.0525 (13)	0.0448 (12)	0.0281 (10)	0.0011 (10)	0.0085 (9)	0.0047 (9)
O2	0.0444 (13)	0.107 (2)	0.0385 (13)	−0.0222 (15)	0.0024 (10)	0.0066 (14)
N1	0.0354 (16)	0.0245 (15)	0.0280 (15)	0.000	0.0076 (13)	0.000
N2	0.0364 (16)	0.0283 (16)	0.0272 (15)	0.000	0.0089 (13)	0.000
C1	0.0259 (13)	0.071 (2)	0.0310 (14)	−0.0051 (13)	0.0104 (11)	0.0042 (14)
C2	0.0409 (16)	0.080 (3)	0.0311 (15)	−0.0024 (17)	0.0043 (12)	0.0064 (16)
C3	0.0416 (17)	0.088 (3)	0.0336 (16)	−0.0069 (19)	0.0047 (13)	−0.0062 (18)
C4	0.0392 (16)	0.074 (2)	0.0447 (18)	−0.0076 (17)	0.0169 (14)	−0.0093 (18)
C5	0.0455 (19)	0.068 (3)	0.0399 (18)	0.0016 (15)	0.0091 (15)	0.0059 (15)
C6	0.0368 (16)	0.074 (3)	0.0302 (15)	−0.0021 (14)	0.0025 (12)	0.0062 (14)
C7	0.070 (3)	0.080 (3)	0.066 (3)	−0.010 (2)	0.023 (2)	−0.014 (2)
C8	0.0342 (15)	0.067 (2)	0.0492 (18)	0.0037 (15)	0.0146 (13)	0.0072 (16)
C9	0.0321 (13)	0.0426 (16)	0.0417 (16)	0.0027 (12)	0.0084 (12)	0.0068 (13)
C10	0.073 (2)	0.0307 (15)	0.0411 (16)	0.0028 (15)	0.0315 (16)	−0.0034 (13)
C11	0.069 (2)	0.0292 (14)	0.0374 (15)	0.0012 (14)	0.0306 (15)	0.0021 (12)
C12	0.0284 (16)	0.0253 (17)	0.0245 (16)	0.000	0.0048 (13)	0.000
C13	0.0260 (16)	0.0236 (17)	0.0259 (16)	0.000	0.0047 (13)	0.000
C14	0.0510 (17)	0.0298 (13)	0.0289 (13)	−0.0056 (12)	0.0207 (12)	−0.0053 (11)
C15	0.0506 (16)	0.0293 (14)	0.0320 (13)	−0.0068 (12)	0.0198 (12)	−0.0030 (11)

### Geometric parameters (Å, °)

**Table:**

Cd1—N1	2.243 (3)	C4—C5	1.393 (5)
Cd1—O1W	2.253 (2)	C4—C7	1.495 (6)
Cd1—O1W^i^	2.253 (2)	C5—C6	1.371 (5)
Cd1—N2^ii^	2.259 (3)	C5—H5A	0.9300
Cd1—O1	2.267 (2)	C6—H6A	0.9300
Cd1—O1^i^	2.267 (2)	C7—H7A	0.9600
S1—C1	1.769 (4)	C7—H7B	0.9600
S1—C8	1.809 (4)	C7—H7C	0.9600
O1—C9	1.266 (4)	C8—C9	1.532 (4)
O1W—H1WA	0.8200	C8—H8A	0.9700
O1W—H1WB	0.805 (17)	C8—H8B	0.9700
O2—C9	1.232 (4)	C10—C11	1.375 (4)
N1—C10^i^	1.325 (3)	C10—H10A	0.9300
N1—C10	1.325 (3)	C11—C12	1.381 (3)
N2—C15	1.343 (3)	C11—H11A	0.9300
N2—C15^i^	1.343 (3)	C12—C11^i^	1.381 (3)
N2—Cd1^iii^	2.259 (3)	C12—C13	1.475 (5)
C1—C2	1.391 (5)	C13—C14^i^	1.389 (3)
C1—C6	1.398 (5)	C13—C14	1.389 (3)
C2—C3	1.367 (7)	C14—C15	1.376 (4)
C2—H2A	0.9300	C14—H14A	0.9300
C3—C4	1.389 (6)	C15—H15A	0.9300
C3—H3A	0.9300		
			
N1—Cd1—O1W	93.87 (6)	C6—C5—H5A	119.2
N1—Cd1—O1W^i^	93.87 (6)	C4—C5—H5A	119.2
O1W—Cd1—O1W^i^	172.26 (12)	C5—C6—C1	121.0 (3)
N1—Cd1—N2^ii^	180.0	C5—C6—H6A	119.5
O1W—Cd1—N2^ii^	86.13 (6)	C1—C6—H6A	119.5
O1W^i^—Cd1—N2^ii^	86.13 (6)	C4—C7—H7A	109.5
N1—Cd1—O1	91.48 (5)	C4—C7—H7B	109.5
O1W—Cd1—O1	90.67 (9)	H7A—C7—H7B	109.5
O1W^i^—Cd1—O1	89.13 (9)	C4—C7—H7C	109.5
N2^ii^—Cd1—O1	88.52 (5)	H7A—C7—H7C	109.5
N1—Cd1—O1^i^	91.48 (5)	H7B—C7—H7C	109.5
O1W—Cd1—O1^i^	89.13 (9)	C9—C8—S1	114.1 (2)
O1W^i^—Cd1—O1^i^	90.67 (9)	C9—C8—H8A	108.7
N2^ii^—Cd1—O1^i^	88.52 (5)	S1—C8—H8A	108.7
O1—Cd1—O1^i^	177.04 (10)	C9—C8—H8B	108.7
C1—S1—C8	105.40 (18)	S1—C8—H8B	108.7
C9—O1—Cd1	123.9 (2)	H8A—C8—H8B	107.6
Cd1—O1W—H1WA	109.5	O2—C9—O1	126.0 (3)
Cd1—O1W—H1WB	123 (3)	O2—C9—C8	118.2 (3)
H1WA—O1W—H1WB	105.7	O1—C9—C8	115.9 (3)
C10^i^—N1—C10	116.8 (4)	N1—C10—C11	123.4 (3)
C10^i^—N1—Cd1	121.62 (18)	N1—C10—H10A	118.3
C10—N1—Cd1	121.62 (18)	C11—C10—H10A	118.3
C15—N2—C15^i^	116.8 (4)	C10—C11—C12	120.2 (3)
C15—N2—Cd1^iii^	121.58 (18)	C10—C11—H11A	119.9
C15^i^—N2—Cd1^iii^	121.58 (18)	C12—C11—H11A	119.9
C2—C1—C6	117.7 (4)	C11^i^—C12—C11	116.0 (4)
C2—C1—S1	126.2 (3)	C11^i^—C12—C13	122.02 (18)
C6—C1—S1	116.1 (3)	C11—C12—C13	122.02 (18)
C3—C2—C1	120.6 (4)	C14^i^—C13—C14	116.2 (3)
C3—C2—H2A	119.7	C14^i^—C13—C12	121.89 (17)
C1—C2—H2A	119.7	C14—C13—C12	121.89 (17)
C2—C3—C4	122.4 (3)	C15—C14—C13	120.4 (3)
C2—C3—H3A	118.8	C15—C14—H14A	119.8
C4—C3—H3A	118.8	C13—C14—H14A	119.8
C3—C4—C5	116.8 (4)	N2—C15—C14	123.0 (3)
C3—C4—C7	120.6 (4)	N2—C15—H15A	118.5
C5—C4—C7	122.6 (4)	C14—C15—H15A	118.5
C6—C5—C4	121.5 (4)		

Symmetry codes: (i) −*x*, *y*, −*z*+1/2; (ii) *x*, *y*+1, *z*; (iii) *x*, *y*−1, *z*.

### Hydrogen-bond geometry (Å, °)

**Table:**

*D*—H···*A*	*D*—H	H···*A*	*D*···*A*	*D*—H···*A*
O1W—H1WA···O2	0.82	1.94	2.667 (4)	148
O1W—H1WB···O1^iv^	0.805 (17)	2.04 (2)	2.782 (3)	154 (4)

Symmetry codes: (iv) *x*, −*y*, *z*+1/2.
